# Clinical outcomes and optical performance of a new segmental refractive extended depth-of-focus intraocular lens

**DOI:** 10.1186/s12886-024-03586-4

**Published:** 2024-08-01

**Authors:** David Rua Amaro, Eckart Bertelmann, Christoph von Sonnleithner

**Affiliations:** Department of Ophthalmology, Charité Medical University, Campus Virchow, Augustenburger Platz 1, 13353 Berlin, Germany

**Keywords:** Cataract, Presbyopia, Intraocular Lens, EDOF

## Abstract

**Background:**

The visual performance and the optical quality of a segmental refractive extended depth-of-focus (EDoF) intraocular lens (IOL) were evaluated in a prospective, single-arm, single-center study.

**Methods:**

A total of 20 patients (40 eyes) after bilateral implantation of the refractive segmental EDoF Acunex Vario AN6V were included. Assessment procedure: refraction outcome, monocular and binocular uncorrected (UCVA) and distance-corrected visual acuity (DCVA), defocus curve, contrast sensitivity (CS), higher-order aberrations (HOAs) and patient satisfaction were evaluated 1 and 3 months after surgery.

**Results:**

At 3-month follow-up, mean spherical equivalent was − 0.23 ± 0.32 D. Binocular uncorrected distance visual acuity (VA) at distance, intermediate and near was − 0.08 ± 0.06 logMAR at 4 m, -0.03 ± 0.06 logMAR at 66 cm and 0.16 ± 0.06 logMAR at 40 cm, respectively. The binocular defocus curve showed a VA better than 0.20 logMAR over a range from + 1.50 to − 2.45 D. Contrast sensitivity aligned with monofocal lenses, highlighting the lens’s clinical value. Aberrometry showed minimal changes in corneal aberrations. NEI-RQL-42-Questionnaire showed a high patient satisfaction for daily activities and revealed reduced dependence on glasses, particularly for near and intermediate vision. The Acunex Vario AN6V demonstrated minimal dysphotopsias, making it a promising option for those seeking spectacle independence.

**Conclusion:**

This segmental refractive EDoF IOL provides a high degree of spectacle independence for far and intermediate distances with functional near VA. Although inducing residual coma aberrations, it showed subjectively good results with little dysphotopsias.

**Trial registration:**

This study protocol was reviewed and approved by ethics committee of Charité University, Berlin, Germany, with approval number EA4/126/20.

## Background

Monofocal lenses are the most implanted intraocular lenses (IOLs) due to their excellent visual performance with minimal disturbing side effects [1]. However, patients´ desire to reduce refractive error and dependency of spectacles has been increasing with longer life expectancy and greater physical health and activity. Over the past decades, the development of multifocal intraocular lenses (mIOLs) incorporating diffraction or refraction principles has offered individuals satisfactory vision at both near and far distances, albeit with some compromise in intermediate vision [2]. Modern activities involving handheld devices and computers commonly necessitate users to rely on their intermediate and near vision. In this context, the development of presbyopia-correcting IOLs is of growing interest [3]. 

Extended depth-of-focus (EDoF) lenses aim to supply an uninterrupted range of vision. They were introduced in 2014 with Tecnis Symfony ZXR00 being the first developed EDoF IOL based on an achromatic diffractive design [4]. Since then, various optical principles have been devised. Most recently, the American Academy of Ophthalmology Task Force Consensus Statement has established the minimum performance criteria for classifying an EDoF-IOL [5]. These lenses should possess an elongated focal range that extends from far distance to intermediate distance, ensuring exceptional visual acuity (VA) for both distance and intermediate vision.

Regarding the IOL optical design, zonal refractive lenses have separate regions that refract light in different ways [6]. This can be achieved either with concentric disk portions, as Mini Well IOL, or with wedged regions, such as the original Hoffer Split Bifocal IOL. This asymmetrical multifocal IOL was introduced in 1982 and initially manufactured by Iolab. In 1990, the first implantation of this lens demonstrated the brain’s ability to simultaneously process two different retinal images imposed on the macula [7]. This IOL configuration was later adapted in 2010 for the Mplus IOL (Oculentis GmbH and Topcon Europe BV) and subsequently for the SBL-3 IOL (Lenstec, Inc. St. Pete Beach, FL), which received FDA approval in 2022 [8, 9]. 

**Fig. 1:**
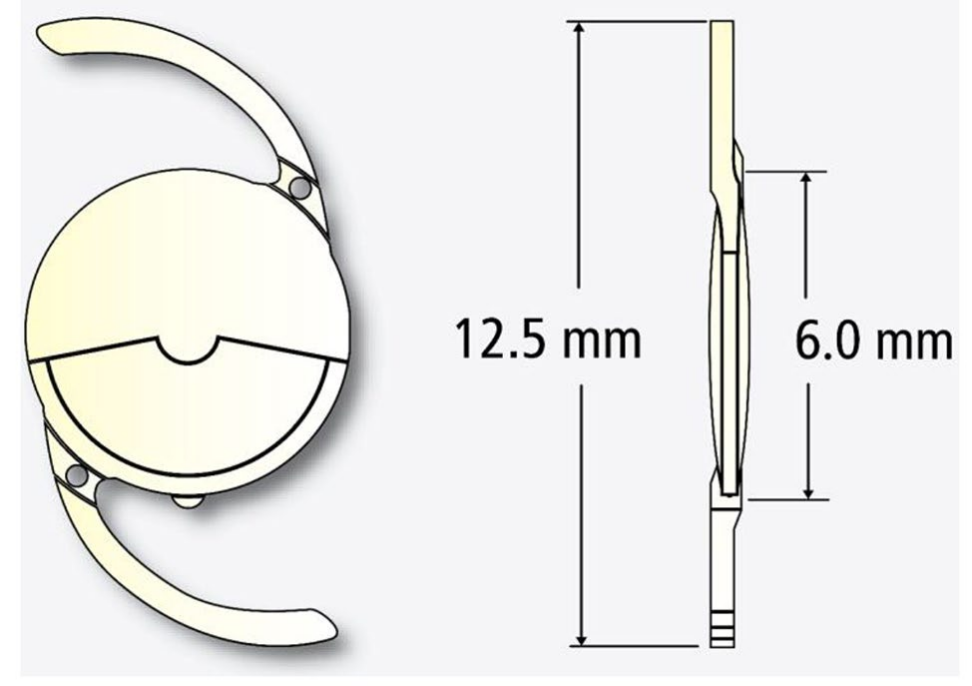
Schematic image of the segmental refractive EDoF IOL (Acunex Vario AN6V, Teleon Surgical)

Reducing the power of addition in segmental refractive lenses allows the conception of zonal refractive EDoF lenses [6]. The Acunex Vario AN6V is a hydrophobic refractive IOL featuring a + 1.50 D addition in an asymmetric near segment, achieving with multiple foci an in vivo EDoF effect (Fig. 1). This study assesses visual performance, depth of focus, aberrometry, and patient satisfaction after bilateral IOL implantation.

## Methods

### Study design

In this prospective study, 20 patients (40 eyes) underwent bilateral phacoemulsification with the novel Acunex Vario refractive extended depth of focus (EDoF) lens at Charité University, Berlin, Germany, between February 2021 and July 2022. Follow-up assessments were conducted at 1 and 3 months post-surgery.

Inclusion criteria comprised individuals with bilateral cataract necessitating surgery, having otherwise unimpaired ocular function, and anticipating a postoperative corrected distance visual acuity (CDVA) of at least 0.20 logMAR. Exclusion criteria involved patients with expected postoperative astigmatism exceeding 1.0 D, a pupil diameter less than 5 mm, history of ocular trauma, prior ocular surgeries, or ocular pathologies affecting visual outcomes.

Preoperative biometry analysis utilized the IOL Master 700 (v. 1.90.12.05) for measurements of corneal power, axial length, and anterior chamber depth (ACD). IOL diopter power for emmetropia (0.0 D) was calculated using Barrett Universal II for all IOLs. In cases where Barrett Universal II was not applicable or yielded inconclusive results, we employed Haigis, SRK/T, or alternative AI-based formulas for greater accuracy and reliability.

Surgeries were performed by two equally skilled surgeons (E.B. and C.v.S.) with a one-week interval between the first and second eye surgeries. Phacoemulsification employed a standard 2.2 mm clear corneal incision under peribulbar anesthesia using the Centurion Vision System from Alcon Laboratories, Inc. Following surgery, patients were given prescribed eye drops and ointment containing cortisone and broad-spectrum antibiotics.

### Postoperative assessment

Patients underwent a comprehensive ophthalmological evaluation at one and three months post the second-eye surgery. This included slitlamp examination, tonometry, and fundoscopy. Visual outcomes were assessed during follow-up visits, and specific evaluations such as defocus curves, aberrometry, and patient satisfaction surveys were conducted at the three-month mark. All assessments were consistently performed by the same examiner (D.R.A.).

Postoperative VA was measured using the 100% contrast Early Treatment Diabetic Retinopathy Study (ETDRS) chart under photopic conditions, presented in logMAR units. Monocular and binocular uncorrected and corrected distance visual acuities (UDVA and CDVA) were tested at 4 m. Additionally, monocular and binocular uncorrected intermediate visual acuity (UIVA) and distance-corrected intermediate visual acuity (DCIVA) were assessed at 66 centimeters. Uncorrected near visual acuity (UNVA) and distance-corrected near visual acuity (DCNVA) were measured at 40 centimeters.

Contrast sensitivity (CS) was evaluated using the Functional Acuity Contrast Test (F.A.C.T.) under different lighting conditions and spatial frequencies: photopic (85 cd/m²), mesopic (3 cd/m²), and mesopic conditions with glare (1 lx). The spatial frequencies tested were 1.5, 3, 6, 12, and 18 cycles per degree (cpd).

Higher-order aberrations (HOAs) were examined through wavefront aberrometry (iTrace, Tracey Technologies, v. 6.2.1) with varying pupil sizes (5, 4, 3 and 2 mm). The surgical impact on these aberrations was analyzed by comparing pre- and post-surgery root mean square (RMS) values.

Monocular and binocular corrected distance defocus curves were generated in 0.50 D increments from + 1.50 D to − 4.0 D defocus at 4 m. The defocus curve was performed with distance-corrected measurements to offer a clear understanding of the IOL’s performance without the variability introduced by uncorrected refractive errors. Patient satisfaction was assessed using the National Eye Institute Refractive Error Quality of Life-42 (NEI-RQL-42) questionnaire [10]. 

### Intraocular lens

The Acunex Vario AN6V IOL is made of a hydrophobic glistening-free acrylate material with additional Ultraviolet-absorbing blue light filter. This foldable one-piece IOL has a biconvex optic design, featuring an anterior sector-shaped intermediate vision segment with a + 1.50 D addition, as well as an aspherical posterior surface. The overall diameter of the IOL measures 12.50 mm, and it is designed with C-loop haptics and an optical zone of 6.0 mm.

### Statistical analysis

Statistical analyses used Microsoft Excel 2016 (v. 16.75.2, Microsoft Corp.) and SPSS Statistics (v.25, IBM Corp.). Data included demographic features, visual and refractive outcomes, and questionnaire results, presented as mean ± SD and (range).

## Results

### Demographic factors and visual outcomes

The mean age of the patients was 61.0 ± 10.6 years (43.0 to 73.0 years), and 55.0% (11 out of 20) were female. The average IOL power measured + 20.50 ± 2.00 D (16.5 to 24.0 D). Refractive outcomes post-surgery showed the following results: at 1 month, the mean sphere was − 0.18 ± 0.33 D and the cylinder was − 0.42 ± 0.38 D. By 3 months, the mean sphere had improved to -0.07 ± 0.33 D, and the cylinder to -0.37 ± 0.37 D. These results indicate a trend toward improved refractive stability over time. Spherical equivalent (SE) improved from − 1.94 ± 2.41 D (-7.25 to 1.50 D) before surgery to -0.23 ± 0.32 D (-1.00 to 0.50 D) three months after second-eye surgery. SE precision of ±0.50 D three months postoperatively was accomplished in 87.5% (35 of 40 eyes). The refractive cylinder reduced slightly from − 0.51 ± 0.44 D preoperatively to -0.37 ± 0.37 D postoperatively.

**Fig. 2:**
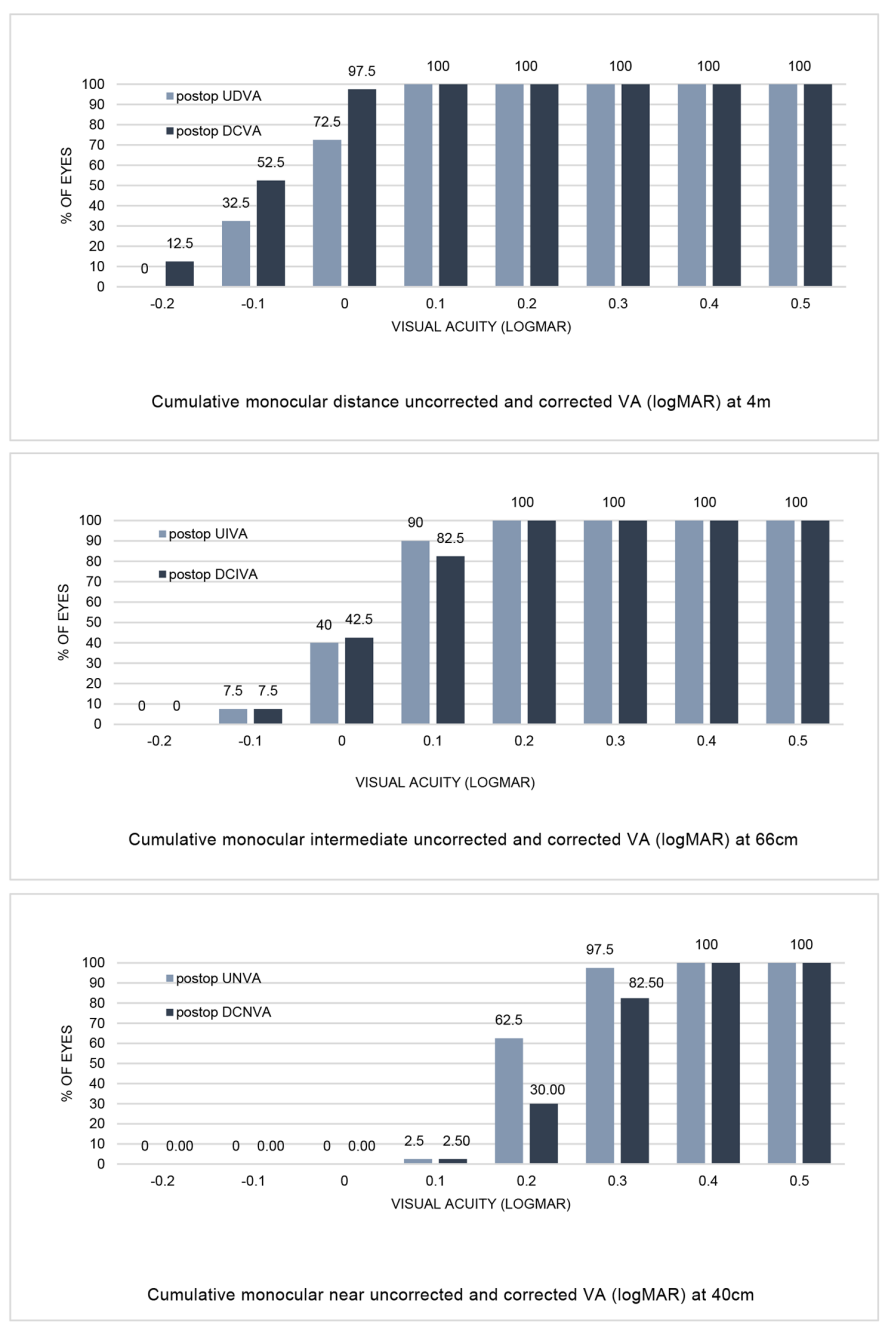
Cumulative monocular VA after implantation of the EDoF IOL (Acunex Vario) at 3 months postoperatively. The percentage (%) of eyes is shown that achieve a certain cumulative uncorrected VA and DCVA, expressed in logMAR. **A**, Cumulative monocular VA at 4 m (*n* = 40 eyes). **B**, Cumulative monocular VA at 66 cm (*n* = 40 eyes). **C**, Cumulative monocular VA at 40 cm (*n* = 40 eyes). VA = visual acuity; DCVA = distance-corrected visual acuity

Table 1 provides details on both monocular and binocular VA at 1 and 3 months post-surgery. Cumulative monocular UCVA and DCVA at various distances three months postoperatively can be found in Fig. 2. Twenty-nine (72.5%) eyes achieved a monocular UDVA of 0.00 logMAR or better, and thirty-nine (97.5%) eyes exhibited a monocular DCVA of at least 0.00 logMAR. All eyes demonstrated both monocular UDVA and DCVA of 0.10 logMAR or higher. At 66 cm, every eye attained a UCIVA of 0.20 logMAR or better, with thirty-six (90.0%) achieving 0.10 logMAR, and sixteen (40.0%) eyes achieving 0.00 logMAR. Regarding DCIVA, thirty-three (82.5%) eyes achieved 0.10 logMAR. At 40 cm, thirty-nine (97.5%) eyes demonstrated a UNVA of 0.30 logMAR or better, and twenty-five (62.5%) exhibited a DCNVA of at least 0.20 logMAR.

**Table 1:** Postoperative visual acuity at 1 Month and 3 Months

Measurement	Monocular (*n* = 40 eyes)	Binocular (*n* = 20 patients)
1 Mo postop		
UDVA 4 m	0.01 ± 0.08	-0.07 ± 0,05
CDVA 4 m	-0.05 ± 0.07	-0.10 ± 0,05
UIVA 66 cm	0.06 ± 0.07	-0.02 ± 0.06
DCIVA 66 cm	0.10 ± 0.07	0.05 ± 0.06
UNVA 40 cm	0.25 ± 0.08	0.20 ± 0.05
DCNVA 40 cm	0.32 ± 0.07	0.29 ± 0.05
3 Mo postop		
UDVA 4 m	-0.04 ± 0.09	-0.08 ± 0.06
CDVA 4 m	-0.09 ± 0.06	-0.13 ± 0.06
UIVA 66 cm	0.03 ± 0.07	-0.03 ± 0.06
DCIVA 66 cm	0.03 ± 0.09	-0.02 ± 0.06
UNVA 40 cm	0.20 ± 0.09	0.16 ± 0.06
DCNVA 40 cm	0.25 ± 0.08	0.21 ± 0.06

Data are reported as mean ± SD

**Fig. 3:**
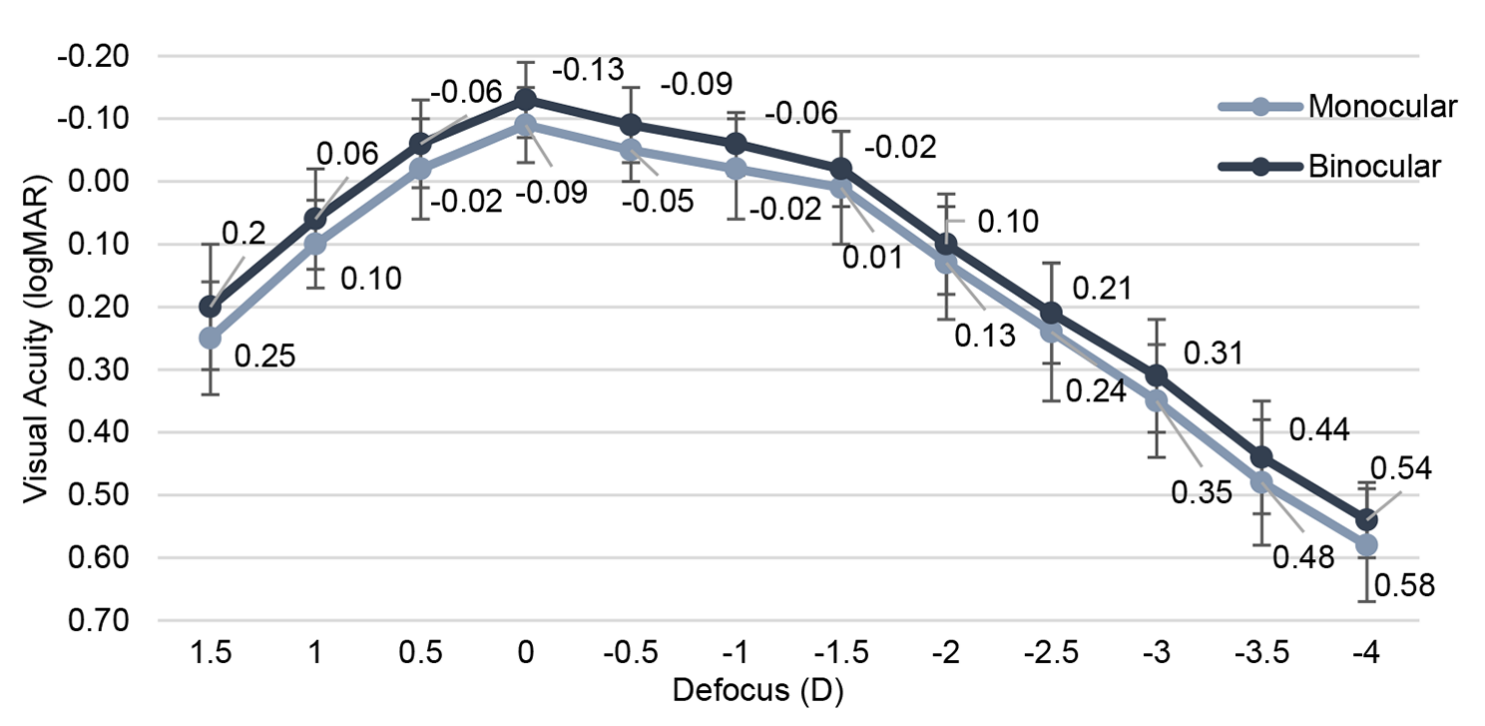
Monocular and binocular distance-corrected defocus curves given in logMAR after implantation of AN6V at 3 months postoperatively (*n* = 20 eyes)

Monocular and binocular distance-corrected defocus curve are depicted in Fig. 3. Optimal outcomes are observed in both curves within the range of 0.50 to -1.50 D, where curves reach a plateau. Both curves exhibit close parallel alignment, although the VA of the binocular defocus curve slightly surpasses that of the monocular curve.

No postoperative complications or posterior capsular opacification were observed throughout the duration of the follow-up.

### Contrast sensitivity

**Fig. 4:**
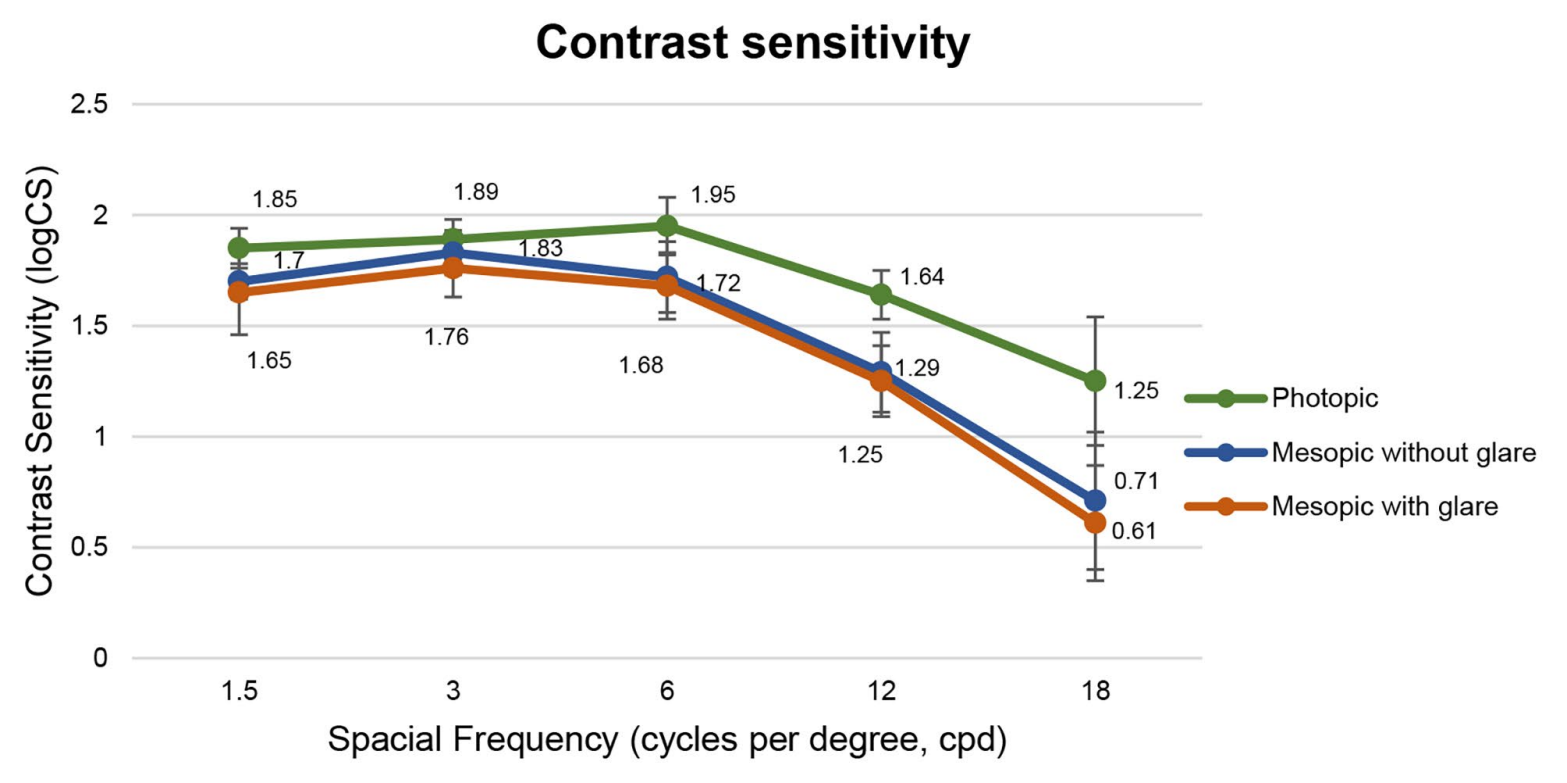
Mean monocular contrast sensitivity photopic, mesopic without glare and mesopic with glare, at 3 months postoperatively

Contrast sensitivity (CS) data are visually represented in Fig. 4. As anticipated, CS was diminished under mesopic conditions, particularly at lower spatial frequencies. The obtained outcomes fell within physiological range established for the measuring apparatus, aligning with values typical of individuals of similar age.

### Aberrometry

**Fig. 5:**
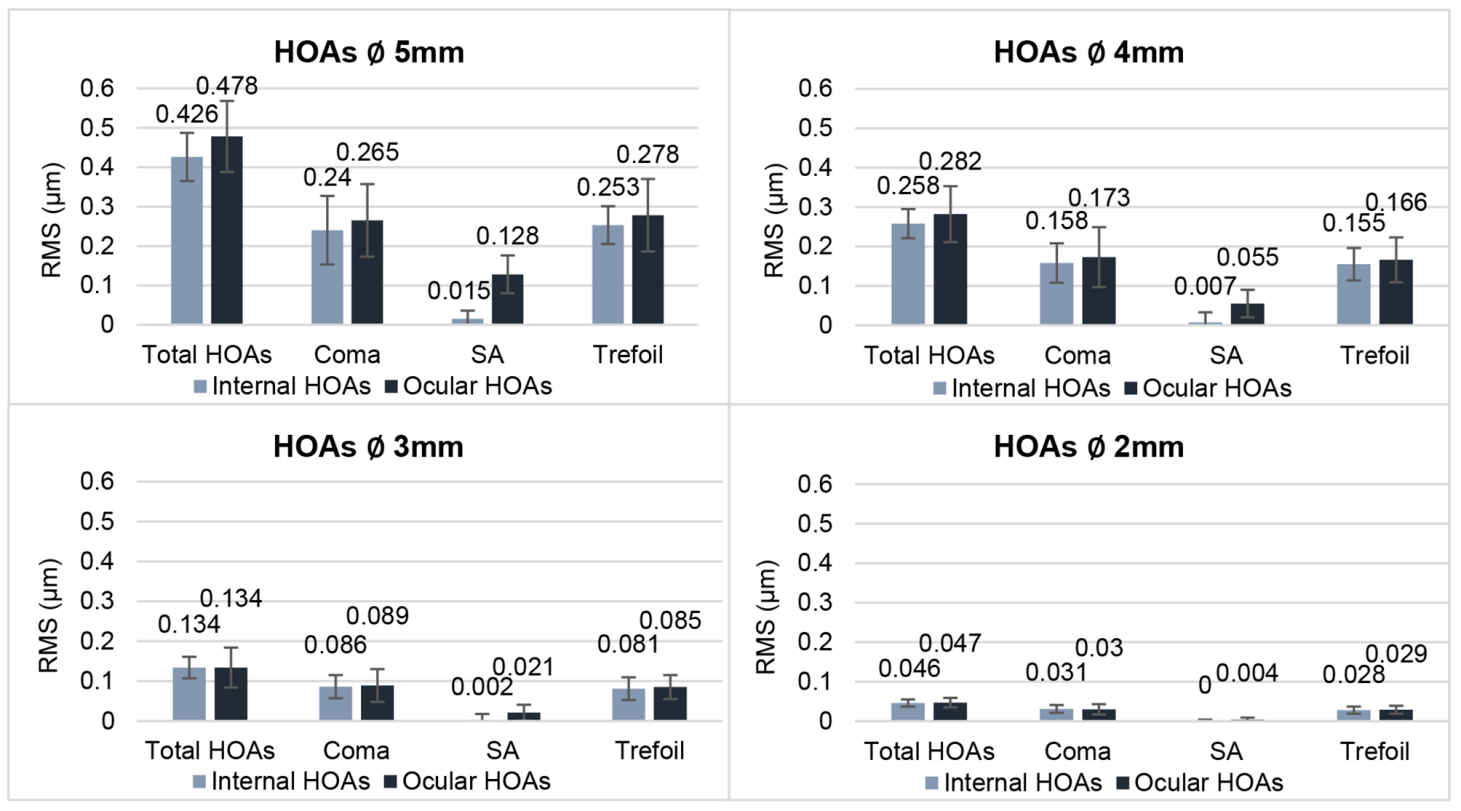
Internal and ocular HOAs at different pupil diameters at the 3-month follow-up. HOA = higher-order aberration; RMS = root mean square; SA = spherical aberration

Results of internal and ocular HOAs can be found in Fig. 5. Primary SA were close to zero. With decreasing pupil diameter, Acunex Vario showed declining values of HOAs. Total HOA in a 3 mm pupil changed from 0.059 ± 0.025 μm to 0.060 ± 0.033 μm by the end of follow-up period. Corneal aberrations were equivalent before and after surgery in 5, 4, 3 and 2 mm pupil diameters.

### Patient satisfaction

**Fig. 6:**
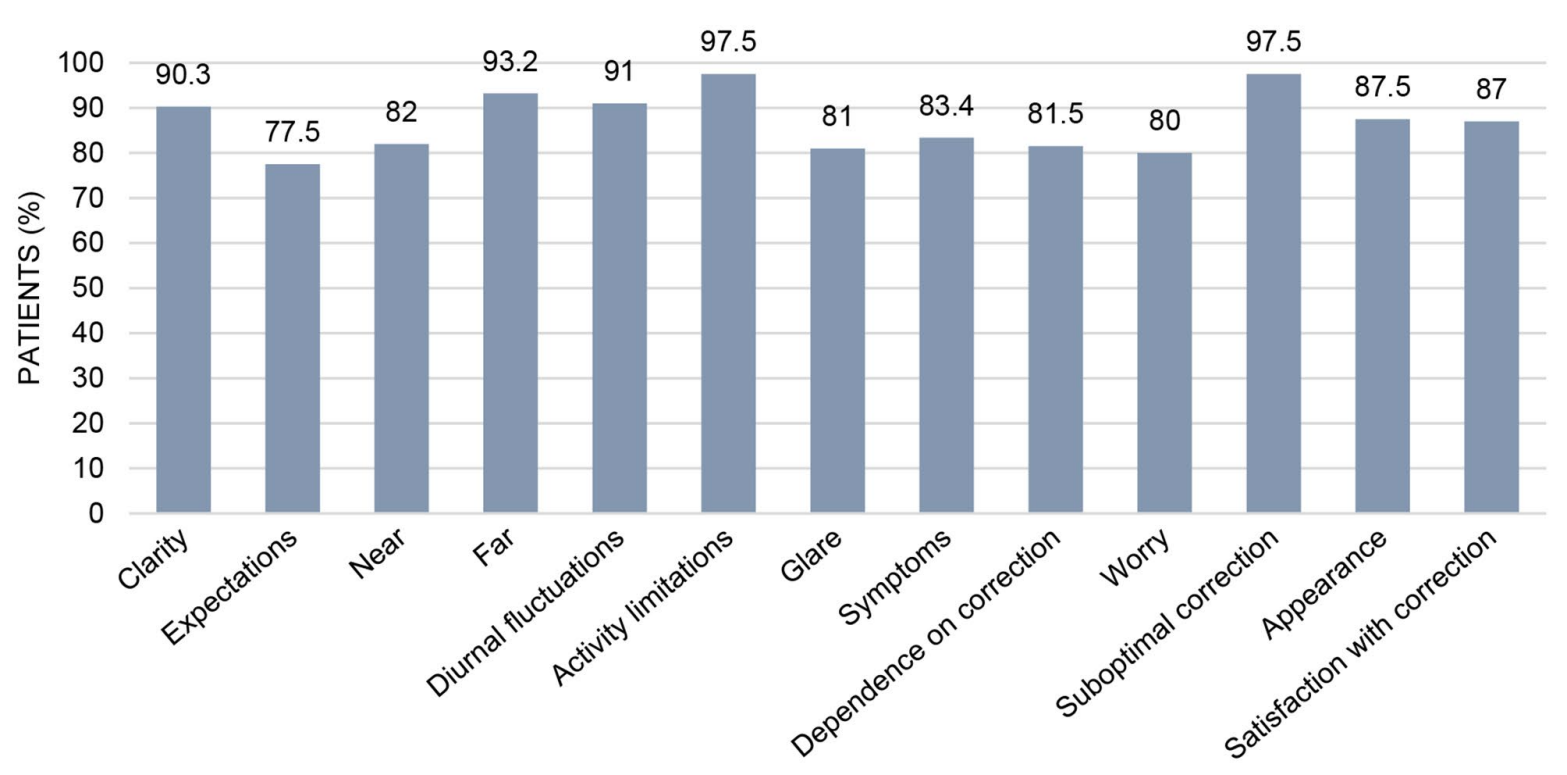
Results of the NEI-RQL-42-Questionnaire (high values indicate favorable result). The Y-axis represents the percentage of satisfied patients. NEI-RQL-42 = National Eye Institute Refractive Error Quality of Life-42

All patients completed the National Eye Institute Refractive Error Quality of Life-42 (NEI-RQL-42) satisfaction questionnaire at the 3-month follow-up. The results demonstrated notable levels of reduced dependence on eyeglasses, particularly for near and intermediate vision (depicted in Fig. 6). Fifteen patients (75.0%) reported either infrequent or no necessity for glasses while reading brief text. In the category “expectations,” overall satisfaction with correction after surgery was approximately 87.0%. Optical phenomena such as halos or starbursts were reported by three patients (15.0%). Four patients (20.0%) reported glare, although all indicated it was not distressing or disabling.

## Discussion

In recent years, various models of presbyopia-correcting IOLs have emerged, enhancing intermediate vision beyond monofocal and multifocal IOLs with fewer dysphotopsias and minimal loss of contrast sensitivity compared to conventional multifocal IOLs [11]. Our study assesses the visual performance and optical quality of a segmental refractive EDoF IOL.

The Acunex Vario IOL qualifies as an EDoF-IOL extending far focus to include the intermediate range, delivering satisfactory distance and intermediate vision [5]. We found favorable unaided monocular and binocular UDVA and corrected CDVA. To our knowledge, there is no literature comparing the AN6V with other monofocal or multifocal IOLs.

Comparable distance monocular VA was achieved from our study group with monofocal IOLs such as Tecnis ZCB00 (-0.05 ± 0.06 logMAR and − 0.14 ± 0.09 logMAR) and Mini 4 IOL (-0.06 ± 0.06 logMAR) [12–14]. The hydrophilic Lentis Comfort LS-313 MF15 IOL, similar in design to the Acunex Vario, demonstrated equivalent visual performance, with Kretz et al. reporting UDVA of 0.00 logMAR and CVDA of -0.08 logMAR in 60 eyes of 30 patients [15]. The diffractive EDoF Tecnis Symfony ZXR00 shows UDVA ranging from 0.08 to 0.01 logMAR and our group found UDVA of -0.02 logMAR with this lens [12, 16]. Greve et al. reported UDVA of 0.04 logMAR and CDVA of -0.02 logMAR with the nondiffractive EDoF IOL Mini Well, noting superior CDVA in the monofocal IOL Mini 4 [13]. 

The + 1.50 D addition of the AN6V IOL provides favorable intermediate vision outcomes and effective near functional UNVA. Corrected distance VA is preferred for comparing IOL visual function due to refractive error [17]. Song et al. found no significant difference in DCIVA between Tecnis Symfony ZXR00 and LS-313 MF15 at 80 cm [18]. In our study, UNVA was better than DCNVA due to a minimal residual myopic error. Despite the better near visual performance, similar refractive mIOLs with + 3.0 D addition power show remarkably poorer intermediate VA than AN6V [19, 20]. Reducing add power improves intermediate VA, enabling functional near vision < 0.50 logMAR, enough to read bigger print sizes [15, 21]. The ANV6 IOL´s defocus curve demonstrated commendable VA across a broad spectrum, covering intermediate vision without discontinuities. Binocularly, a visual acuity of 0.20 logMAR or better was achieved within + 1.50 to -2.45 D (monocularly, from + 1.33 to -2.32 D), covering a range of 3.95 D (or monocularly, 3.65 D).

The contrast sensitivity results were comparable to those of monofocal lenses studied by our group [12–14]. Several studies highlight the exceptional contrast vision of refractive segmental IOLs, establishing their equivalence with monofocal IOLs [22]. In contrast, diffractive EDoF designs reduce effective light energy reaching each focal plane, resulting in loss in contrast sensitivity [5, 23]. 

Slightly higher residual coma aberrations could be attributed to the IOL’s asymmetric refractive power distribution. Alió et al. found similar increases with the Lentis Mplus LS-312, a segmental refractive mIOL with + 3 D add, showing nearly 0.500 μm of coma aberrations [22]. Elevated primary coma, although causing optical blur, can paradoxically extend the depth of focus, enhancing near VA [24]. Internal SA were nearly neutral, resulting in slightly positive ocular SA, which can increase depth of focus without impairing the distance vision [25]. 

Our objective VA outcomes aligned with high spectacle independence scores in the NEI-RQL-42 questionnaire. Subjective performance of the IOL was over 90% for distance and above 80% for near vision. However, 70% of the patients had at least some difficulty reading small print and 85% wore spectacles at least occasionally or prolonged reading. Comparisons between trifocal and bifocal IOLs reveal similar quality of life and satisfaction outcomes, with trifocal lenses generally providing better near VA [11, 26–28]. General satisfaction appears to correlate more with intermediate vision than with near vision, suggesting that intermediate distances are crucial for modern tasks like using computers, mobile phones and tablets [28]. 

Patient-reported photopic phenomena, including halos and glare, were comparable to our previous reports with monocular IOLs. The AN6V achieved a 80% of positive rating in the glare category, compared to 60–65% with the Tecnis ZCB00 and 70% with the Mini 4 IOL [12–14]. Dysphotopsia is an inherent challenge with mIOLs due to the overlap of multiple images on the retina caused by redirection of light to various focal points [29]. Diffractive technology, like the ZXR00 IOL, have higher disturbances [30]. Our study with this IOL reached only 50% patient satisfaction for glare [12]. Nondiffractive EDOFs, such as the Alcon Vivity DFT015 and Mini Well IOL show similar patterns to aspheric monofocal IOLs [31, 32]. As the Acunex Vario also lacks diffractive optics, it might cause fewer dysphotopsias. Song et al. found milder photopic symptoms with segmental refractive LS-313 MF15 IOL compared to the the ZXR00 IOL [18]. Despite slightly higher residual coma aberrations, our patients did not report increased perception of glare or halos in the dark, with 80% of patients experiencing no difficulties driving at night.

The study´s limitations include a limited sample size and potential selection bias due to the COVID-19 pandemic. The 3-month follow-up period, although common in studies, does not provide insights into long-term outcomes, especially regarding neuroadaptation. Studies suggest that while optical phenomena might be detectible after years, the subjective perception of visual disturbances tends to decrease over time [33]. 

When selecting an IOL, thorough preoperative assessments and discussions about potential advantages and disadvantages are essential [6]. A blended vision approach, such as targeting slight myopia in the non-dominant eye, can enhance binocular visual outcomes and reduce the risk of dysphotopsias compared to multifocal IOLs [34]. For small deviations from emmetropia, such as -0.50 D, the mini monovision approach has been successful with EDoF IOLs like Tecnis Symfony [4, 35]. Breyer at al. showed improved binocular UNVA and UIVA without impairment of UDVA using the LENTIS Comfort LS-313 MF 15 with a target of -1.50 D in the nondominant eye [3]. These findings suggest that blended vision with EDoF IOLs, such as AN6V, is an effective alternative to trifocal IOL implantation.

In conclusion, the Acunex Vario AN6V demonstrated an extensive range of vision up to 60 cm, with high postoperative satisfaction and minimal visual disturbances. This EDoF IOL is a promising choice for patients seeking freedom from spectacles for both distant and intermediate distances. However, its suitability for eyes that have undergone previous refractive surgery should be considered, and a preoperative assessment of HOAs is recommended.

## Conclusion

In summary, our study supports the Acunex Vario IOL’s classification as an EDoF IOL, aligning with the AAO’s consensus. The lens effectively provides satisfactory distance and intermediate vision, comparable to traditional monofocal IOLs.

The + 1.50 D addition of the Acunex Vario IOL yields favorable outcomes for intermediate and near vision, showcasing commendable visual acuities across a broad defocus spectrum. Contrast sensitivity results align with monofocal lenses, emphasizing the clinical value of refractive segmental IOLs.

Post-surgery, corneal aberrations remained consistent, with slightly higher residual coma attributed to the IOL’s design. Subjective outcomes indicate high patient satisfaction, with minimal reported photic disturbances such as halos and glare.

In conclusion, the Acunex Vario IOL emerges as a promising choice, offering enhanced visual performance, particularly in intermediate vision, and demonstrating overall patient satisfaction, making it a valuable option in the realm of presbyopia-correcting intraocular lenses.

## Data Availability

The datasets used and/or analysed during the current study are available from the corresponding author on reasonable request.

## Abbreviations

AAO: American Academy of Ophthalmology
ACD: Anterior Chamber Depth
CS: Contrast Sensitivity
DCIVA: Distance-Corrected Intermediate Visual Acuity
DCNVA: Distance-Corrected Near Visual Acuity
DCVA: Distance-Corrected Visual Acuity
EDoF: Extended Depth-of-Focus
ETDRS: Early Treatment Diabetic Retinopathy Study
F.A.C.T.: Functional Acuity Contrast Test
HOAs: Higher-Order Aberrations
IOL: Intraocular Lens
IOL Master: Optical Biometry Device
logMAR: Logarithm of the Minimum Angle of Resolution
mIOLs: Multifocal Intraocular Lenses
NEI-RQL-42: National Eye Institute Refractive Error Quality of Life-42
RMS: Root Mean Square
SA: Spherical Aberration
SD: Standard Deviation
SE: Spherical Equivalent
UDVA: Uncorrected Distance Visual Acuity
UIVA: Uncorrected Intermediate Visual Acuity
UNVA: Uncorrected Near Visual Acuity
VA: Visual Acuity
